# VLP‐factory™ and ADDomer^©^: Self‐assembling Virus‐Like Particle (VLP) Technologies for Multiple Protein and Peptide Epitope Display

**DOI:** 10.1002/cpz1.55

**Published:** 2021-03-17

**Authors:** Duygu Sari‐Ak, Joshua Bufton, Kapil Gupta, Frederic Garzoni, Daniel Fitzgerald, Christiane Schaffitzel, Imre Berger

**Affiliations:** ^1^ Department of Medical Biology, School of Medicine University of Health Sciences Istanbul Turkey; ^2^ Bristol Synthetic Biology Centre BrisSynBio University of Bristol Bristol United Kingdom; ^3^ School of Biochemistry, Biomedical Sciences University of Bristol Bristol United Kingdom; ^4^ Imophoron Ltd, St. Philips Central St. Philips Bristol United Kingdom; ^5^ Geneva Biotech SARL Genève Switzerland; ^6^ School of Chemistry University of Bristol Bristol United Kingdom; ^7^ Max Planck Bristol Centre for Minimal Biology University of Bristol Bristol United Kingdom

**Keywords:** antigenic epitope, baculovirus expression vector system (BEVS), immunization, MultiBac, protein and peptide display, vaccine, virus‐like particle (VLP)

## Abstract

Virus‐like particles (VLPs) play a prominent role in vaccination as safe and highly versatile alternatives to attenuated or inactivated viruses or subunit vaccines. We present here two innovations, VLP‐factory™ and ADDomer**^©^**, for creating VLPs displaying entire proteins or peptide epitopes as antigens, respectively, to enable efficient vaccination. For producing these VLPs, we use MultiBac, a baculovirus expression vector system (BEVS) that we developed for producing complex protein biologics in insect cells transfected with an engineered baculovirus. VLPs are protein assemblies that share features with viruses but are devoid of genetic material, and thus considered safe. VLP‐factory™ represents a customized MultiBac baculovirus tailored to produce enveloped VLPs based on the M1 capsid protein of influenza virus. We apply VLP‐factory™ to create an array of influenza‐derived VLPs presenting functional mutant influenza hemagglutinin (HA) glycoprotein variants. Moreover, we describe MultiBac‐based production of ADDomer^©^, a synthetic self‐assembling adenovirus‐derived protein‐based VLP platform designed to display multiple copies of pathogenic epitopes at the same time on one particle for highly efficient vaccination. © 2021 The Authors.

This article was corrected on 20 July 2022. See the end of the full text for details.

**Basic Protocol 1**: VLP‐factory™ baculoviral genome generation

**Basic Protocol 2**: Influenza VLP array generation using VLP‐factory™

**Basic Protocol 3**: Influenza VLP purification

**Basic Protocol 4**: ADDomer^©^ BioBrick design, expression, and purification

**Basic Protocol 5**: ADDomer^©^ candidate vaccines against infectious diseases

## INTRODUCTION

Recombinant protein production is an essential prerequisite for cost‐efficient and versatile production of complex biologics which can be used as scaffolds to display proteins and peptides that can represent antigenic epitopes from pathogens including viruses that cause infectious diseases. Viruses are characterized by a proteinaceous shell encasing and protecting the viral genetic material that is released and replicated upon infection of the host cell organism. The immune system of the host typically mounts its defense against pathogenic surface structures representing antigenic epitopes with the objective to defeat and clear out the pathogen. Early on, it was recognized that administering such antigenic epitopes, for example in the form of heat‐inactivated viral pathogen species, can result in protective responses setting the stage for vaccinations‐arguably the most successful intervention in terms of saving human lives to date. An attractive alternative to administering inactivated or attenuated virus derives from recombinant production of the proteinaceous shell, or parts of it, resulting in virus‐ like particles (VLPs). VLPs can be physically similar or even identical to live virus, but devoid of genetic information and thus safe, as replication cannot occur. VLPs are used for vaccination against a range of infectious diseases including influenza and viral infection−based malignancies such as cervical cancer caused by papilloma virus (Charlton & Lua, 2017; Cox & Hollister, 2000; Jeong & Seong, 2017; Pouyanfard & Müller, 2017; Schiller & Lowy, 2006; Temchura & Überla, 2017).

*Escherichia coli* is by and large the dominating system for recombinant protein expression in molecular biology; however, complex biologics such as VLPs often cannot be expressed efficiently in *E. coli* due a plethora of reasons, including post‐translational modifications or specialized folding machineries not present in *E.coli* but which eukaryotic expression systems can afford ( Nettleship et al., 2015; Nettleship, Assenberg, Diprose, Rahman‐Huq, & Owens, 2010; Nie et al., 2009; Nie et al., 2016; Vijayachandran et al., 2011).

The baculovirus expression vector system (BEVS) has emerged as a particularly powerful technology for the production of complex protein biologics that are authentically targeted and post‐translationally modified including disulfide‐bond formation and glycosylation. We developed MultiBac, a modular baculovirus‐based expression system particularly suited for efficiently producing challenging protein assemblies for a wide range of applications, as we and others have described in considerable detail (e.g., Barford, Takagi, Schultz, & Berger, 2013; Berger et al., 2013; Berger, Fitzgerald, & Richmond, 2004; Bieniossek, Imasaki, Takagi, & Berger, 2012; Bieniossek, Richmond, & Berger, 2008; Fitzgerald et al., 2006; Fitzgerald et al., 2007; Nie et al., 2009; Sari et al., 2016; Trowitzsch, Bieniossek, Nie, Garzoni, & Berger, 2010; Trowitzsch, Palmberger, Fitzgerald, Takagi, & Berger, 2012; Vijayachandran et al., 2011). MultiBac has been widely adopted in academia and industry, accelerating research and development world‐wide, with the focus in recent years shifting to customizing the system for specialized tasks including, for example, humanized glycoprotein production (Palmberger, Klausberger, Berger, & Grabherr, 2013; Palmberger, Wilson, Berger, Grabherr, & Rendic, 2012), genetic code expansion (Koehler et al., 2016), or multifunctional DNA delivery in mammalian and human cells and tissues, among others (Aulicino, Capin, & Berger, 2020; Mansouri et al., 2016; Sari et al., 2016).

**Figure 1:**
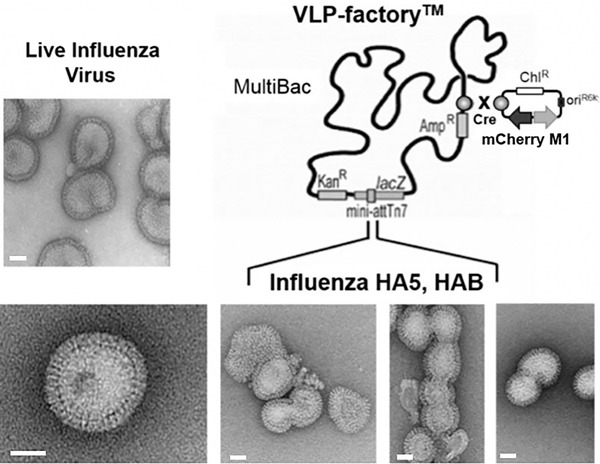
MultiBac‐Based VLP‐factory™. On the right, the MultiBac baculoviral genome customized for enveloped virus‐like particle production is shown schematically. The capsid‐forming influenza H1N1 matrix protein M1 and the fluorescent protein mCherry are integrated into the viral backbone. The mCherry protein is a reporter for tracking virus performance. Influenza surface protein (hemagglutinin HA and/or neuraminidase NA) insertion into this VLP‐factory™ by means of transfer plasmids results in baculoviruses producing influenza virus‐like particles (VLPs). Electron micrographs of a selection of the VLPs is shown below. Recombinant influenza VLPs produced by the customized MultiBac genome (VLP‐factory™) and live influenza virus are close to identical in terms of size, shape, and appearance (inset, image courtesy of R. Ruigrok). Scale bars (50 μm) are drawn in white (adapted from Sari‐Ak et al., 2019).

In this article, we describe VLP‐factory™, a customized MultiBac system that is tailored for high‐level expression of VLP based on influenza M1 capsid protein. A gene encoding M1 from H1N1 influenza strain, as well as a gene encoding mCherry fluorescent protein for monitoring the course of virus amplification and VLP production in the cell culture, were integrated into the viral backbone (Fig. 1). An array of influenza VLPs including sets of mutations in a potentially immune‐suppressive domain (ISD) within the influenza surface protein hemagglutinin (HA) were efficiently produced using VLP‐factory™. ISDs were discovered in retroviruses, and evidence suggests that influenza HA also comprises an ISD overlapping with its fusion peptide segment, potentially impacting on innate immune responses in macrophages and dendritic cells (Bahrami, Laska, Pedersen, & Duch, 2016; Holm et al., 2012). The ISD is located on the structurally sensitive part of HA, which is critical for membrane fusion. Hence, any design in this part that would abolish immune suppression and enhance antigenicity would need to be carefully crafted to maintain HA functionality in membrane fusion. To identify such mutants, an array of influenza VLPs containing randomized amino acids at certain positions in the ISD were required (Sari‐Ak et al., 2019). Resulting influenza VLP variants (wild‐type HA or HA ISD mutants) can be functionally tested for membrane fusion activity in vitro as well as immune‐suppressive activity in vivo in animal models. It would be very useful to have influenza VLPs containing ISD mutations for use as VLP‐based hyper‐immunogenic antigens that could represent strongly and broadly protecting influenza vaccines. HA‐comprising ISD mutations identified as most potent in abolishing immune suppression and increasing HA antigenicity will likely elicit stronger neutralizing antibody titers upon administration as compared to unmodified HA. The process of preparing M1‐based influenza VLPs comprising HA with a series of ISD mutations is detailed here. By the same token, other viral or non‐viral glycoproteins, domains thereof, or structured epitopes or target proteins of choice can be displayed efficiently using VLP‐factory, by providing a native or heterologous transmembrane domain to anchor the proteins to be displayed on the enveloped VLP surface.

Transfer plasmid generation and insertion into the MultiBac genome are detailed in our previous publications (Bieniossek et al., 2008; Fitzgerald et al., 2006; Haffke, Viola, Nie, & Berger, 2013). Therefore, in this article, we focus on the process of production, purification, and analysis of the influenza VLP array using VLP‐factory™, our customized MultiBac‐derived baculoviral expression tool. Influenza M1 protein is the key element of VLP‐factory™. We identified M1 from the H1N1 influenza strain as particularly suited for self‐assembly and capsid formation (Sari‐Ak et al., 2019). Moreover, capsids formed by H1N1 M1 are readily released from the cells in the expression culture, acquiring the lipid bilayer envelope comprising heterologously expressed membrane proteins during the budding process. Because of these characteristics of H1N1 M1, our VLP‐factory™ is not limited to flu VLPs with HA in the envelope, but can be used to produce VLPs displaying diverse viral envelope proteins, one or several simultaneously, from diverse influenza subtypes or even from other pathogens, that are expressed from genes inserted in addition to M1 and mCherry into the VLP‐factory™ baculovirus. These proteins will then be successfully integrated in the cell membrane and thus into the envelope of M1‐based VLPs during budding, as shown here for HA. VLP‐factory™, thus, has considerable potential as a flexible production tool for a wide range of VLP vaccines displaying surface protein antigens of viral pathogens.

Non‐enveloped VLPs lacking a lipid membrane likewise represent popular candidates for new and better vaccines (Bachmann et al., 2013; Draper, et al., 2010; Frietze, Peabody, & Chackerian, 2016; Fuenmayor, Gòdia, & Cervera, 2017; Unzueta et al., 2015). A major drawback of enveloped VLPs, but also of many protein‐only VLPs currently available, and in fact of most vaccines currently deployed, originates from their lack of thermostability, necessitating an elaborate cold‐chain from production to point‐of‐care to maintain refrigeration, which is often difficult or even impossible in poor and remote areas, leading to significant losses (Ashok, Brison, & LeTallec, 2016).

**Figure 2:**
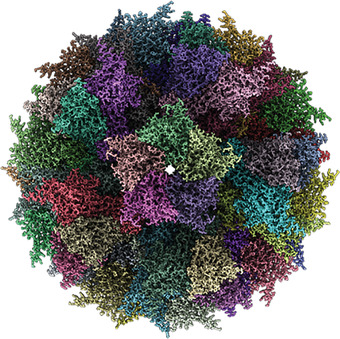
The ADDomer^©^. Electron cryo‐microscopy (Cryo‐EM) map of the synthetic self‐assembling ADDomer^©^ particle, formed by 60 identical protomers. The protomers assemble into 12 pentons, forming a dodecahedron characterized by remarkable thermostability (Vragniau et al., 2019).

To overcome these limitations, we developed ADDomer^©^, our synthetic self‐assembling multiepitope peptide display scaffold for highly efficient vaccination (Vragniau et al., 2019) (Fig. 2). ADDomer^©^ is derived from a component of human adenovirus, the so‐called penton base protein. Adenoviruses are widely used in gene therapy and considered safe (Crystal, 2014). The adenovirus capsid consists of hexons, pentons, and fiber extrusions. Intriguingly, the penton‐forming base protein of some adenovirus serotypes can spontaneously self‐assemble into a symmetric particle consisting of 60 protomers (Norrby, 1966). These protomers assemble into 12 pentons forming a dodecahedron, preserving adenovirus‐like ability to penetrate epithelial cells (Fender, Boussaid, Mezin, & Chroboczek, 2005). Exploiting these characteristics, we developed ADDomer^©^, our thermostable synthetic dodecahedron platform for highly efficient multiepitope display, as a next‐generation VLP platform for vaccines and protein therapeutics (Vragniau et al., 2019). ADDomer^©^ is produced at high levels using our MultiBac baculovirus expression system. A wide range of epitopes can be displayed using ADDomer, including, to date, epitopes as short as a few amino acids or encompassing several hundred amino acid residues, potentially including structured domains (Vragniau et al., 2019).

**Figure 3:**
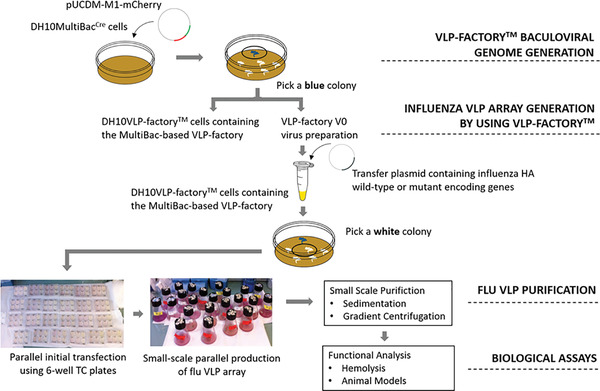
Overview of VLP‐factory™ protocol. VLP‐factory™ was created for the expression of a functional influenza VLP variant assay. A transfer plasmid library including genes encoding wild‐type and mutant influenza HA5 and HAB proteins was transformed into VLP‐factory™ (Basic Protocol 1). Illustrated is the initial virus in 6‐well plates, already giving the signal for the expression of mCherry that we perceive from the red color change (Basic Protocol 2). Parallel expression enables extraction and purification of the influenza VLPs by sedimentation and gradient centrifugation for functional studies (Basic Protocol 3).

This article presents detailed instructions for generating, by utilizing VLP‐factory™, an influenza VLP array displaying mutants of the HA glycoprotein. Moreover, this article presents the MultiBac‐based approach of the Ad3‐derived ADDomer^©^ for displaying multiple peptide epitopes. Basic Protocol 1 provides instructions for VLP‐factory baculoviral genome generation. Basic Protocol 2 outlines stages to prepare the influenza VLP Array by using VLP‐factory™. Basic Protocol 3 describes purification of influenza VLPs. The different parts of the project are illustrated in a workflow diagram (Fig. 3). Basic Protocol 4 details how to design, produce, and purify ADDomer^©^ using the MultiBac system. Finally, in Basic Protocol 5, the approach to engineer the ADDomer^©^ platform for vaccine development against an infectious disease is explained.

## STRATEGIC PLANNING

It is critical to plan in advance all the procedures in detail to successfully produce VLPs with the MultiBac baculovirus/insect cell expression system. For gene insertion using the transfer plasmids of the MultiBac system, we recommend planning all steps of the cloning process in silico via suitable software at the beginning of all multiprotein complex expression projects.

Some of the gene expression cassettes are integrated by site‐specific recombination using Cre recombinase. We recommend planning this part carefully beforehand using our published protocols (Bieniossek et al., 2008; Fitzgerald et al., 2006).

An objective of the influenza VLP array produced by the VLP‐factory™ is to identify mutants that maintain the HA membrane fusion feature while simultaneously inactivating the immune suppressive activity, with the aim to induce stronger immune responses by the resulting VLP vaccine candidates. We describe here the protocol for production of the influenza VLP variants (wild‐type HA or HA ISD mutants). A particular challenge regarding execution of this protocol resides in the many baculoviruses produced in parallel for the expression for different ISD mutants, requiring the utmost care to avoid cross‐contamination during cell infection.

## Basic Protocol 1 VLP‐FACTORY™ BACULOVIRAL GENOME GENERATION

The influenza M1 and HA subunits and the functional mutant−encoding genes are designed in silico using a DNA cloning software of choice (i.e., VectorNTI, ApE, others). and are then inserted into transfer plasmids of choice called Donor and Acceptor (the MultiBac transfer plasmids available are listed in Table 1 to be used based on the individual experimental design). In addition to HA, further influenza protein−encoding genes (NA, M2) can be likewise inserted, resulting in multivalent VLPs. Polycistronic expression cassettes can be prepared using a variety of different methods‐total DNA synthesis, restriction/ligation cloning, ligation‐independent cloning (LIC), sequence and ligation−independent cloning (SLIC), or Gibson cloning (Casini, Storch, Baldwin, & Ellis, 2015) ***‐*** as described in detail previously (Benoit et al., 2016; Celie, Parret, & Perrakis, 2016). The technique used is entirely up to what the individual user feels most comfortable with. Given the recent decrease in costs incurred as a result of the competition among the commercial suppliers, we recommend using custom DNA synthesis (importantly including complete cassette sequencing also of promoters and terminators), which will make expression cassette construction easier and faster, in particular for generation of mutant arrays, as we carried out here for influenza HA.

**Table 1:** MultiBac Donor and Acceptor Plasmids

Vector names	Host strain	Replicon	Recombination and multiplication sides	Antibiotic resistance genes (1000× stock concentration)
***Acceptor plasmids***				
pFL	TOP10^*a*^	ColE1	Tn7L, Tn7R, *loxP*, multiplication module M	Ampicillin (100 mg/ml) Gentamycin (10 mg/ml)
pKL	TOP10	pBR322	Tn7L, Tn7R, *loxP*, multiplication module M	Kanamycin (50 mg/ml) Gentamycin (10 mg/ml)
***Donor plasmids***				
pUCDM	BW23474^*b*^	R6Kγ	*loxP*, multiplication module M	Chloramphenicol (30 mg/ml)
pSPL	BW23474	R6Kγ	*loxP*, multiplication module M	Spectinomycin (50 mg/ml)
***Other plasmids***				
MultiBac bacmid	DH10MultiBac DH10MultiBac^Cre^	F1	Tn7L, Tn7R, *loxP*	Kanamycin (50 mg/ml) Ampicillin (100 mg/ml)
pBADZ‐HisCre	TOP10 DH10MultiBac^Cre^	ColE1	None	Zeomycin (50 mg/ml) (Zeocin)

^*a*^ *recA^‐^ endA^‐^ pir^‐^* other cloning strains can be used here as well.

^*b*^ *recA^‐^ endA^‐^ pir^+^* other pir gene expressing strains can be used here as well.

In this protocol, the plasmid used is created by custom gene synthesis for DNA encoding M1 from H1N1 influenza virus and hemagglutinin derived from both H1N1 and H5N1 influenza viral strains. We have also used commercial mutagenesis services to insert carefully planned potential immune‐modulating mutations into the hemagglutinin encoding genes.

In the in silico design step, it is important to eliminate internal restriction sites, as this can be important for subcloning into the MultiBac plasmids when we need to insert any other proteins such as NA and M2, or glycosylases to modulate the sugar structure of the heterologous proteins (Palmberger & Rendic, 2015; Palmberger et al., 2012; Palmberger et al., 2013) for our parallelized expression experiment set‐up.

Codon optimization is another key point to be applied for best expression results of the gene(s) of interest in insect cells, which is done via the web‐based algorithms offered by most of the synthetic DNA suppliers (e.g., http://www.idtdna.com/CodonOpt; or OptimumGene™, from http://www.genscript.com), typically free of charge. This procedure will also eliminate potentially harmful RNA secondary structure elements in the transcripts.

In this section, MultiBac based VLP‐factory™ production will be described. First, the preparation of baculoviral genome for the expression of virus‐like particles with the genes inserted into viral backbone encoding M1 from H1N1 influenza strain and mCherry fluorescent protein for monitoring virus performance and VLP production is detailed. Then, transformation into DH10MultiBac cells comprising the MultiBac baculoviral genome is described, and finally DH10VLP‐factory™ cells are prepared.

All reagents are prepared using ultrapure water (Millipore Milli‐Q system or equivalent; conductivity of 18.2 MΩ·cm at 25°C) and analytical grade reagents. All the buffers, antibiotics and enzymes are stored at –20°C.

### Materials

An empty Donor plasmid (pUCDM is the plasmid used here and for which the steps below are written) for the insertion of genes encoding influenza H1N1 M1 protein (GenBank ID ABD59883.1) and fluorescent protein mCherry (GenBank ID ANO45948.1) via multiple cloning sites (Fitzgerald et al., 2006)
Restriction endonucleases and reaction buffers (NEB)
T4 DNA ligase and buffer (NEB cat. no. M0202)
*E. coli* competent cells containing *pir* gene (for Donor plasmids): e.g., BW23473 or BW23474 strains (Invitrogen)
Agar for pouring plates
Media (LB, TB, SOC; see Current Protocols article: Elbing & Brent, 2018)
Antibiotics (1000× stock): ampicillin (100 mg/ml), kanamycin (50 mg/ml), chloramphenicol (30 mg/ml), tetracycline (10 mg/ml)
QIAquick plasmid purification kit (Qiagen, cat. no. 27106)
*E.coli c*ompetent cells DH10MultiBac harboring MultiBac baculoviral genome (Fitzgerald et al., 2006).
0.1 M CaCl_2_ (Sigma Aldrich, cat. no. C1016); sterilize by autoclaving
80% (v/v) glycerol (Sigma Aldrich, cat. no. G5516); sterilize by autoclaving
Liquid nitrogen
Cre recombinase enzyme and buffer (NEB cat. no. M0298)
Isopropyl β‐d‐1‐thiogalactopyranoside IPTG (Sigma Aldrich cat. no. I6758).
BluoGal (Sigma Aldrich cat. no. B2904)
Isopropanol
70% ethanol
Insect cell medium (e.g., SF900 II SFM; ThermoFisher) to grow insect cells (cat. no. 10902088)
Sf21 cells (ECACC 05030202) or Sf9 (ATCC CRL‐1711) insect cells growing in culture
Transfection reagent (e.g., FuGENE from Promega, cat. no. E2311; JetPEI from Polyplus Transfection, cat. no. 101‐10N; or X‐tremeGENE from Merck, cat. no. 6366236001)

6‐well tissue culture plates; VWR, cat. no. 10062‐892
100 × 15 mm petri dishes
Pipettes and pipette tips
Spectrophotometer to determine OD_600_
Refrigerated centrifuge
42°C water bath or heat block for heat shock
26°C incubator
15‐ml Falcon tubes

Additional reagents and equipment for preparing V_0_ virus (see Current Protocols article: Biennosek, Richmond, & Berger, 2008, Basic Protocol 5) and agarose gel electrophoresis (see Current Protocols article: Voytas, 2001)

1 Insert the genes encoding influenza H1N1 M1 protein (GenBank ID ABD59883.1) and fluorescent protein mCherry (GenBank ID ANO45948.1) into the multiple cloning sites MCS1 and MCS2 of plasmid pUCDM (Fitzgerald et al., 2006) under polh and p10 promoter control, respectively, for generating pUCDM‐M1‐mCherry. Use generic restriction enzyme/ligation‐based cloning or ligation‐independent methods (Casini et al., 2015; Haffke et al., 2013) according to user preference.
2 Transform pUCDM‐M1‐mCherry into pir+ *E.coli* cells expressing the *pir* gene product on which propagation of pUCDM‐based ‘Donor’ plasmids depend (e.g., PIR1 and PIR2 from Invitrogen) due to their conditional origin of replication (Fitzgerald et al., 2006, Metcalf, Jiang, & Wanner, 1994). Plate the transformed cells on LB agar plates (100 × 15 mm size) containing 30 µg/ml chloramphenicol, and incubate at 37°C overnight. Use single colonies from this plate to inoculate 25 ml bacterial culture Luria Broth (LB) medium for growing cells and preparing pUCDM‐M1‐mCherry (Fitzgerald et al., 2006).
3 Prepare 1 µg of pure pUCDM‐M1‐mCherry plasmid by using the QIAquick plasmid purification kit, following the standard plasmid preparation procedure according to the manufacturer's instructions.
4 Prepare DH10MultiBac^Cre^ chemical competent cells containing the MultiBac baculoviral genome and expressing Cre recombinase enzyme (which catalyzes an in vitro Cre‐LoxP reaction for fusing Donor and Acceptor plasmids via their LoxP sites) according to on published procedures (Berger et al., 2004). Briefly:
  - Grow cells in 500 ml sterile LB medium to an OD_600_ of 0.5‐0.6 from an overnight culture.
  - Centrifuge the cells 10 min at 3000 × *g*, 4°C. Resuspend and wash twice, each time with 100 ml of ice‐cold 0.1 M CaCl_2_ at 4°C. Resuspend the final pellet in 5.6 ml of ice cold 0.1 M CaCl_2_ and 2.4 ml of ice‐cold sterile 80% glycerol. Keep everything at 4°C.
  - Make 100‐µl aliquots, flash freeze cells in liquid nitrogen, and finally store at −80°C.
5 Transform pUCDM‐M1‐mCherry plasmid into DH10MultiBac^Cre^ cells by using a standard salt‐dependent transformation protocol. Briefly:
  - Add 500 ng to 1 µg of the plasmid to the competent cells and incubate on ice for 10‐15 min.
  - Subject cells to a heat shock at 42°C for 45‐60 s, and then incubate on ice for 2 min.
  - Add 400‐500 µl of medium (LB, TB, or SOC) and recover for 4‐6 hr or overnight at 37°C.
  - Plate the cells on LB agar plates containing 10 µg/ml tetracycline, 50 µg/ml kanamycin, and 30 µg/ml chloramphenicol antibiotics, 1 mM IPTG, and 100 µg/ml BluOGal color reagent. Incubate at 37°C overnight.
    Several blue colonies should grow on the plate.
6 Select a single blue colony for bacterial culture growth by inoculating it in 4‐5 ml of LB medium. Prepare competent cells and store flash‐frozen (liquid nitrogen) in aliquots at −80°C as described in step 4.
  These are the DH10 VLP‐factory™ cells containing the MultiBac‐based VLP‐factory baculoviral genome (Fig. 1).
7 Prepare VLP‐factory V_0_ virus from a frozen aliquot according to published protocol (see Basic Protocol 5 in Current Protocols article Bieniossek et al., 2008). Briefly:
  - First grow an overnight culture from a frozen cell aliquot from step 6 in 2‐3 ml of LB medium at 37°C shaker.
  - On the next day, spin the cells 10 min at 3000 × *g*, room temperature. By applying the QIAquick plasmid purification kit, the pellet is used to prepare bacmid. Firstly, add 300 µl P1 buffer onto the pellet and pipette the mixture up and down to resuspend it. Then, transfer the mixture into a new 1.5‐ml tube and add 300 µl P2 buffer. Gently invert the tube until the mixture is homogenous, but do not incubate more than 5 min. Add 300 µl N3 buffer to the tube, and a white precipitate would appear at this stage. Invert the tube again gently and then spin it 10 min at 17,000 × g, room temperature. Carefully transfer the supernatant into a fresh tube and spin again for 5 min at 17,000 × *g*, room temperature.
  - Transfer the supernatant into a fresh tube, add 700 µl of isopropanol, and mix gently to homogenize. Spin 10 min at 17,000 × *g*, room temperature. There will be a small DNA pellet at the bottom; remove the supernatant carefully without disturbing the pellet. Add 200 µl of 70% ethanol to the tube carefully, spin 5 min at 17,000 × *g*, room temperature, and remove the supernatant carefully without disturbing the pellet. Add 50 µl of 70% ethanol and bring the tube into the sterile hood.
  - Remove the ethanol and let pellet dry. Add 2 ml insect cell medium to 5 wells in a 6‐well culture plate and 3 ml insect cell medium to the last well to keep it as a cell control. Add 0.5‐1.0 million Sf21 cells in 1 ml volume to the wells drop by drop, evenly, except the cell control well, which will only have medium.
    Sf21 cells should be used from the batch that have been monitored for 2‐3 days before the infection in terms of showing a healthy profile for cell growth. These cells must be maintained daily with a fresh insect cell medium to keep the cell density in the flask ideally in between 0.5 and 1 × 10^6^ cells/ml to ensure optimal growth conditions.
  - Add 30 µl of sterile MilliQ water to the pellet and resuspend the DNA by gently tapping the bottom of the tube. Transfer 10 µl into a fresh tube to check it on an agarose gel (see Current Protocols article: Voytas, 2000). Add 200 µl of insect cell medium to the remaining 20 µl.
  - In a separate fresh tube, add 100 µl of insect cell medium to 10 µl of transfection reagent and mix, then add 100 of this mix to the tube containing the DNA/medium mix from step 7e. Mix gently and add 150 µl of the mix drop‐by‐drop to two wells of the plate with insect cells. Incubate at 26°C for 48‐60 hr without shaking. Cells that are infected with VLP‐factory™ virus will get bigger in size and will turn brightly red due to mCherry expression. Collect the V_0_ virus in sterile 15‐ml Falcon tubes.

## Basic Protocol 2 INFLUENZA VLP ARRAY GENERATION USING VLP‐FACTORY™

Here, DH10VLP‐factory™ cells containing baculoviral genome will be transformed with the transfer plasmid array consisting of wild‐type and mutant influenza HA protein‐encoding genes. Hence, we will have the VLP variants expressed in insect cells that we can visually follow by means of the red color change in the shaker flasks.

### Additional Materials (also see Basic Protocol 1)

An empty Acceptor plasmid for the insertion of genes encoding wild type and mutant influenza HA protein via multiple cloning sites (Fitzgerald et al., 2006)
Erlenmeyer shaker flasks, 250 ml

1 Insert the genes encoding wild‐type and mutant influenza HA proteins into the transfer plasmids (‘Acceptor’, Fitzgerald et al., 2006) as per steps 1‐3 of Basic Protocol 1.
  These acceptor plasmids contain a common ColE1‐derived origin and can be propagated in any E. coli cloning strains such as TOP10, TG1, DH10, HB101.
2 Transform DH10VLP‐factory™ aliquots with transfer plasmids encoding the array made up of wild‐type and mutant influenza HA proteins by using standard a salt‐dependent transformation protocol. Next, plate the cells on agar containing kanamycin, gentamycin, tetracycline, IPTG, and BluOGal.
  Refer to Basic Protocol 1, step 5, substeps a‐d for the above procedures.
3 Pick a single white colony from each transformation and prepare composite VLP‐factory™ bacmids. Use these bacmids to infect insect cells in 6‐well plates and generate V_0_ initial virus.
  The bacmid preparation and V0 initial virus production is done as in step 7 of Basic Protocol 1.
  Already at this early V_0_ stage in 6‐well plates, composite VLP‐factory™ produces clones stained bright red (Fig. 3) from mCherry expression.
  In case the agar plates are aged (agar plates which were prepared more than a week ago as a big batch and stored at 4˚C) or there are many colonies on the plate, we suggest waiting an extra day to observe full blue/white color development, to eliminate false positive selections.
4 Harvest the VLP‐factory™ V_0_ viruses producing influenza VLPs after 48‐60 hr. Amplify to produce V_1_ virus by adding V_0_ viruses to 250‐ml shaker flasks containing 50 ml insect cell culture (Bieniossek et al., 2008). Cell cultures turn red starting from 24‐48 hr post infection. Collect the V_1_ virus at 24 hr post infection.
  We have produced and processed batches of 25 different VLP array members at a time via this approach, to be able to control logistics. In the high‐throughput approaches to culturing insect cells, parallel processing of many more specimens at the same time in smaller volumes in a table‐top multi‐fermenter is possible following established robotics protocols that we developed (Monteiro, Bernal, Chaillet, Berger, & Alves, 2016).

## Basic Protocol 3 INFLUENZA VLP PURIFICATION

In this protocol, the purification method is described for the VLPs expressed using VLP‐factory™. Once purified as described below, functional assays of choice can be carried out in vitro and in vivo.

### Materials

V1 virus (cell culture from Basic Protocol 2, step 4)
Phosphate‐buffered saline (PBS; Thermo Fisher Scientific; cat. no. 10010023)
20% and 60% (w/v) sucrose (Sigma Aldrich; cat. no. S0389) in PBS, autoclaved or sterile filtered using a 20‐μm nylon filter)

Sterile Falcon or Greider tubes (15 ml, 50 ml).
Tabletop centrifuge
Sterile Eppendorf pipettes.
Ultracentrifuge with Beckman SW28 rotor (or equivalent)
38‐ml sterile ultracentrifugation tubes (Beckman).

**Figure 4:**
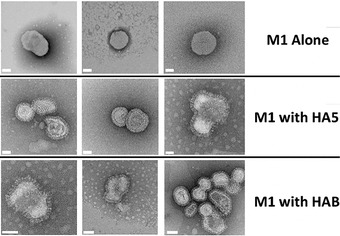
Influenza VLPs. A selection of VLP‐factory™‐produced samples were examined by negative‐stain electron microscopy (EM). M1 expression alone already produces budded enveloped capsids (top row) in the absence of influenza envelope proteins. Co‐expression of influenza wild‐type or mutant HA and HAB results in VLPs displaying the characteristic protruding spike‐shaped pattern (middle and bottom rows). Scale bars (50 μm) are colored in white.

1 Approximately 3 days after proliferation arrest (3 dpa), e.g., when the cell count in the culture remains unchanged, the cell cultures will have a bright red color due to co‐expressed mCherry (Fig. 3). Transfer the cells into a sterile 50‐ml Falcon tube.
2 Spin down cells 3 min at 3000 × *g*, 4°C, in a tabletop centrifuge. It is important to process all the influenza VLP purification steps on ice.
3 Repeat step 1 with a fresh 50‐ml Falcon tube by transferring supernatant to get rid of cell debris that has been carried over.
4 Ultracentrifuge the cleared supernatant overnight at 121,896 × *g*, 4°C, in an SW28 rotor or equivalent, with centrifuge tubes containing 38 ml of supernatant each.
5 Resuspend the pellets by gently mixing with a pipette in 1000 µl PBS.
6 Prepare 38‐ml volume ultracentrifuge tubes with 20% (w/v) sucrose with a cushion of around 2 ml of 60% (w/v) sucrose at the bottom of the tube.
7 Load 1 ml of resuspended VLP pellet (from step 4) on top of the 20% sucrose layer.
8 Prepare an identical tube with the same sucrose cushion for use as balance.
9 Ultracentrifuge the tubes overnight at 121,896 × *g*, 4°C, in an SW28 rotor.
10 Remove 20% sucrose solution by gentle pipetting and collect the white layer between the two sucrose solutions (interface on top of the 60% cushion).
11 Load the collected white layer on another discontinuous 20%‐60% sucrose gradient.
12 Ultracentrifuge the tubes overnight at 121,896 × *g*, 4°C, in an SW28 rotor.
13 Collect the visible white bands containing purified VLP species and store at 4°C for further in vitro use, including EM (Fig. 4).

**Figure 5:**
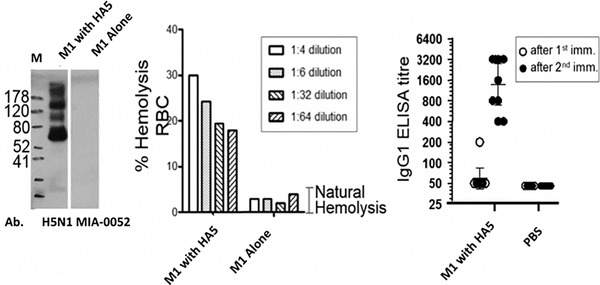
VLP‐factory™‐produced influenza VLPs are functionally active. By Western blotting, the presence of HA protein in HA5 M1 VLP preparation was examined with a specific antibody (H5N1 MIA‐0052) showing the presence of specific HA bands (left). The right lane in the Western blot is the control containing VLPs comprising M1 only, showing no HA signal. M stands for molecular weight marker (sizes sin kDa). Recombinant influenza VLP was analyzed in a hemolysis experiment (middle). The *y* axis denotes the percentage of lysed red blood cells (RBC) in the experiment. The ELISA plot (right) shows antibody titers (IgG1) after two injections (adapted from Sari‐Ak et al., 2019). The *y* axis represents the level of antibody measured in these experiments.

14 To remove sucrose for injection in animal models (Fig. 5) or for other downstream processing (DSP) procedures such as electron microscopy, ultracentrifuge again as described above, then carefully remove the sucrose‐containing supernatant. Resuspend the pellet gently by pipetting up and down in sterile cold PBS (adjust the concentration based on the required particle density for injections).

## Basic Protocol 4 ADDomer^©^ BioBrick DESIGN, EXPRESSION, AND PURIFICATION

The comparison of primary sequences from adenovirus serotypes revealed that two regions in the penton base protein comprise high variability in amino acid sequence and length. These are the variable loop (VL) and arginine‐glycine‐aspartic acid (RGD) loop. The RGD loop is characterized by a conserved tripeptide motif (‐RGD‐) that regulates integrin‐based penetration of the adenovirus into target cells (Gout, Schoehn, Fenel, Lortat‐Jacob, & Fender, 2010).

The low conservation of the VL and RGD loops suggests that one or many antigenic epitopes can be inserted into these regions of the penton base protein. Starting from the Ad3 adenovirus serotype, we thus set out to design a synthetic gene in a “BioBrick” format (Shetty, Endy, & Knight, 2008) providing multiple easily accessible insertion loci for generating protomers comprising epitope sequences or even small protein domains in the VL and RGD loops, optionally in a library format (Vragniau et al., 2019). The crystal structure of the Ad3 protomer (Szolajska et al., 2012) provided guidance for the proper design of the loci based on the secondary structure motifs flanking the loop regions.

The RGD loop in wild‐type Ad3 is significantly larger than the VL, and the RGD motif is critical for cell internalization. We therefore elected to maintain the RGD motif and introduce two insertion loci, before and after the RGD tripeptide motif, respectively. Together with one locus in the VL, we thus engineered our ADDomer**^©^** plug‐and‐play multiepitope display platform comprising three independent epitope insertion sites.

**Figure 6:**
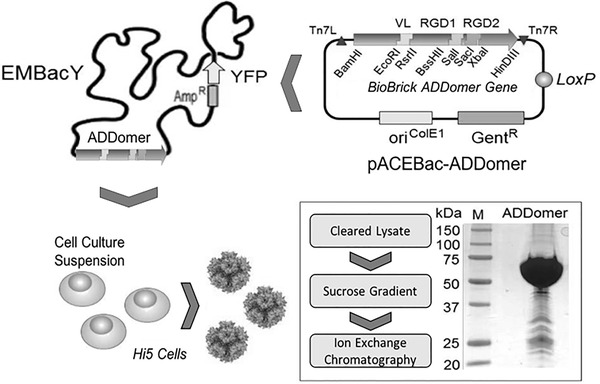
ADDomer^©^ VLP production. MultiBac‐based production of ADDomer^©^. VLP production (Basic Protocol 4) is shown schematically, including a Coomassie‐stained SDS‐PAGE section of highly purified VLP. Plasmid pACEBac‐ADDomer comprises the synthetic gene encoding ADDomer^©^ in a BioBrick format as indicated (adapted from Vragniau et al., 2019). EMBacY is a version of the MultiBac baculovirus comprising a YFP reporter gene in the viral backbone. VL, insertion site engineered in variable loop; RGD1 and RDG2, insertion sites in RGD loop; Tn7L, Tn7R and mini‐attTn7, recognition and attachment sequences for Tn7 transposase; LoxP, site‐specific recombination site for Cre enzyme; Gent^R^, Gentamicin resistance gene; Kan, Kanamycin; Amp, Ampicillin; LacZ, gene for blue/white screening of composite genome; ori^ColE1^, replication origin; YFP, yellow fluorescent protein; M, molecular weight marker (sizes given in kDa).

For high‐level expression of ADDomer**^©^**, we implemented the MultiBac system (Sari et al., 2016; Fig. 6), preparing a pACEBac‐ADDomer plasmid (Table 1) as a generic starting point for all epitope insertions exploiting this scaffold (selected examples shown in Table 2). ADDomer**^©^** was expressed with excellent yield (∼1 g ADDomer from 1 liter of insect cell culture). Quality control of the MultiBac‐produced ADDomer**^©^** sample evidenced negligible nucleic acid or endotoxin contamination (Vragniau et al., 2019).

**Table 2:** ADDomer Epitope Sequences (adapted from Vragniau et al., 2019)

Epitope name^*a*^	Primary sequence	Context
Chikungunya^*b*^	STKDNFNVYKATRPYLAH (Kam et al., 2012)	Human infectious disease
Zika	DAHAKRQTVVVLGSQEGAV (Shawan et al., 2014)	
OVA	SIINFEKL (Villegas‐Mendez et al., 2010)	Human melanoma model
Newcastle	PDEQDYQIRMAKS (Cho et al., 2008)	Livestock infectious disease epitope
Gumboro	MPKTHNSGRSNVDGGGSTLHLPHLWRPLSGGGSHNAKYVSAESWGGSHPDSIHPFLASPGGGSDTLHGHGFTNWF (Wang et al., 2007)	
Extended	ENLFYQSEQGGGGAGGGNNSGSGAEENSNAAAAAMQPVEDMNDHAIRGDTFATRAEEKRAEAEAAAEAAAPAAQPEVEKPQGGSGGSGGAKIEAATAAAEAKANIVASDSTRVANAGEVRGDNFAPTPVPTAESLLADVSEGTDGGSGGSGGTETTTLAVAEETSEDDDITRGDTYITEKQKREAAAAEVKK (Zubieta, Schoehn, Chroboczek, & Cusack, 2005)	For testing (the combined RGD loop sequences from human Adenovirus serotypes Ad2, Ad11 and Ad3, disassociated via flexible linker sequences)

^a^ The given amino acids in the table above include the epitope flanked by linker residues GGSG (N‐term) and GSGG (C‐term) for overall flexibility.

^b^ Chikungunya epitope consists of an N‐terminal linker residue GGSG, and a TEV protease cleavage site right before the epitope (GGSGENLYFQ'S…, and the TEV recognition site underlined, S as the first serine in epitope sequence).

### Materials

Human *Ad3* gene sequence encoding the penton base protein (GenBank Z29487)
Plasmid pACEBac1 (Geneva Biotech SARL, Switzerland; see Table 1)
DH10EMBacY cells (Geneva Biotech SARL, Switzerland)
*Bam*HI and *Hin*dIII restriction enzymes
Hi5 insect cells (ECACC 94‐20‐1) growing in culture
SF900 II SFM (Thermo Fisher) to grow insect cells (cat. no. 10902088)
Bovine serum albumin (BSA; New England Biolabs, cat. no. B9200)
Dimethylsulfoxide (DMSO)
Liquid nitrogen
Hypotonic lysis buffer (see recipe)
Phosphate‐buffered saline (PBS) solution (Thermo Fisher Scientific, cat. no. 10010023)
15% and 40% (w/v) sucrose
Purification buffer gradient solutions (see recipe)

In silico design software (ApE, SnapGene, Vector NTI)
Centrifuge
Gradient Master 107 (BioComp Instruments)
Ultracentrifuge with Beckman SW28 rotor
Sterile ultracentrifugation tubes (Beckman)
6‐8 kDa MWCO dialysis membrane (Spectrum™ Spectra/Por™; cat. no. 132660)
High Q column (Bio‐Rad; cat. no. 7324122)

Additional reagents and equipment for Tn7‐dependent integration into baculoviral genome (see Current Protocols article Bieniossek et al., 2012, Basic Protocol 4), generation of V_0_ virus (Basic Protocol 1, step 7), and generation of V_1_ virus (Basic Protocol 2, step 4), SDS‐PAGE (see Current Protocols article: Gallagher, 2012), and dialysis (see Current Protocols article: Phillips & Signs, 20005)

**Figure 7:**
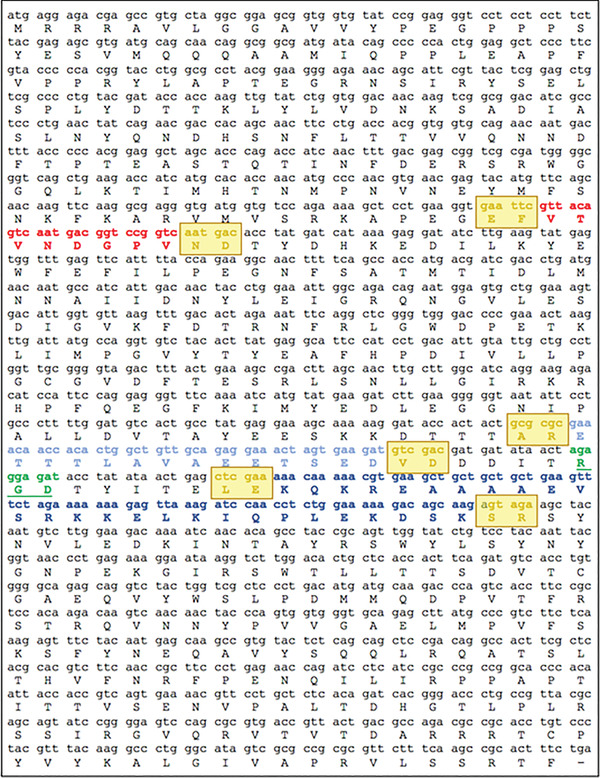
ADDomer^©^ VLP scaffold sequence. Three parts in the protomer region which are evolutionarily nonconserved engineered with BioBrick format into exchangeable cassettes. One cassette (colored in red) is placed within the VL region, while two additional cassettes, RGD1 (colored in light blue) and RGD2 (colored in dark blue), are located within the loop including a functional RGD tripeptide sequence. Amino acid residues encoded by restriction enzymatic sites in BioBrick design are indicated (colored in yellow and boxed). The RGD tripeptide sequence is highlighted (colored in green).

1 Perform in silico design using ApE, SnapGene, or Vector NTI software of a synthetic DNA encoding ADDomer in the BioBrick format with functionalized insertion loci for facile and rapid custom DNA insertion. Make sure to maintain, on the DNA level, the exact restriction enzyme recognition sequences flanking the epitope insertion sites (Fig. 6), thus adhering to the BioBrick design. Importantly, verify.
2 Flank the insertion loci with the unique restriction sites (Fig. 6) that were removed from anywhere else in the Ad3 penton base encoding gene.
  This makes sure that the BioBrick format is adhered to.
3 For the BioBrick design, paste restriction sites flanking the insertion loci in regions devoid of any secondary structure elements (check for secondary structure−devoid region in the structure of penton base protein in PyMOL), maintaining the structural integrity of the penton base protein.
4 After synthesis of the gene encoding ADDomer and codon optimization for recombinant expression in insect cells (offered free of charge nowadays by all DNA synthesis companies: e.g., Genscript, Twist, ThermoFisher, etc.), insert the designed synthetic DNA into pACEBac1 plasmids by digesting the plasmid with *Bam*HI and *Hin*dIII restriction enzymes and carrying out ligation according to standard protocols [see, e.g., New England Biolabs (NEB) catalog at https://international.neb.com/] to yield pACEBac‐ADDomer.
5 Integrate pACEBac‐ADDomer into the EMBacY baculoviral genome via Tn7 transposition (see Current Protocols article Bieniossek et al., 2012, Basic Protocol 4).
  Tn7 transposition happens via Tn7 L and Tn7 R present on the transfer plasmid into the attTn7 site present in the EMBacY baculoviral genome. The primary sequence of the encoded ADDomer is provided (Fig. 7).

6 Based on established protocols, transfect to generate live virions and ADDomer expressed in Hi5 insect cells (Bieniossek et al., 2008).
  Hi5 insect cells should be pre‐adapted to flask and maintained every day to keep the cell density at optimal growth level, 0.5‐1 million cell per ml.
  - Generate V_0_ and V_1_ virus as described in step 7 of Basic Protocol 1 and step 4 of Basic Protocol 2, respectively.
  - Define optimal volume of V_1_ virus needed for infection of insect cells by infecting cells with different volumes of it, and then use that volume to infect insect cells to make baculovirus‐infected insect cells (BIICs).
  - For this, collect 200 ml infected at 1 million cell per ml concentration cells on day of proliferation arrest by centrifuging 10 min at 400‐600 × *g*, 26°C.
  - Resuspend in a filter‐sterilized mix of 18 ml Sf900 medium, 0.2 g BSA, and 2 ml of DMSO. Prepare 1‐ml aliquots in cryovials, place at –20°C for 1 hr, then at –80°C for 24‐48 hr, and then finally store in liquid nitrogen.
  - Use BIICs to do large‐scale expression in Hi5 insect cells by adding 1 alliquot of BIIC to 1 L of insect cells at 1 million cells per ml concentration. Harvest the cells 48‐72 hr post infection by centrifuging 10 min at 1000 × *g*, 4°C, for 10 min and then storing the pellet at −80°C after flash freezing in liquid nitrogen.
7 Resuspend flash‐frozen insect cell pellets which expressed ADDomer in hypotonic lysis buffer and lyse three times via freezing in liquid nitrogen and thawing on ice.
8 Prepare a continuous sucrose gradient by mixing 15% and 40% (w/v) sucrose solutions made in PBS with Gradient Master 107. Clarify lysate from debris via centrifugation for 30 min at 6000 × *g*, 4°C, prior to subjecting the cleared supernatant to a continuous sucrose gradient (15% to 40%) centrifugation (Fender, Ruigrok, Gout, Buffet, & Chroboczek, 1997). Load the lysate on the gradient and ultracentrifuge for 18 hr in Beckman SW 41 rotor at 287,472 × *g*, 4°C.
9 Combine ADDomer‐containing fractions as identified by SDS‐PAGE analysis of the fractions (see Current Protocols article: Gallagher, 2012), which migrate in the gradient at about 30% to 40% sucrose. Dialyze (see Current Protocols article: Phillips & Signs, 2005) the combined fractions against purification buffer using a 6‐8 kDa MWCO dialysis at 4°C overnight.
10 Further purify ADDomer by ion‐exchange chromatography via a High Q column (Bio‐Rad) with 50 mM NaCl purification buffer and applying a linear salt gradient from 50 to 500 mM NaCl purification buffer. Equilibrate the column prior to applying ADDomer with 5 column volumes of purification buffer containing 50 mM NaCl.
11 Identify fractions containing purified ADDomer by tracking absorbance at 280 nm and analyzing fractions by SDS‐PAGE (Gallagher, 2012) These fractions elute between 330 and 400 mM NaCl, which can be conveniently traced by monitoring conductance. Pool fractions comprising ADDomer and store at ambient temperature or frozen (−80°C).
12 Purify additional ADDomer variants of choice designed in step 1 according to the same protocol (Fig. 6). Choose the number of ADDomer variants you wish to purify depending on the availability of the purification equipment in your laboratory and the level of automation.

## Basic Protocol 5 ADDomer^©^ CANDIDATE VACCINES AGAINST INFECTIOUS DISEASES

Infectious diseases continue to constitute major global challenges, forcefully evidenced most recently by the SARS 2 coronavirus outbreak that caused the COVID‐19 pandemic, wreaking havoc world‐wide. Vaccination is uniquely suited to overcome these serious threats. Pre‐COVID‐19, Zika and Chikungunya virus infectious diseases, which are transmitted by mosquitoes, were causing alarm. Originally confined to sub‐Saharan Africa, these diseases are increasingly spreading to the Northern Hemisphere, accelerated by deforestation and global warming. In the Chikungunya virus, the E2EP3 linear peptide has been identified as the major neutralizing epitope, with specific antibodies present in the majority of patients (Kam et al., 2012). The E2EP3 neutralizing epitope is located at the N terminus of the viral E2 glycoprotein, including the first 18 amino acids starting from N‐terminal serine (Fig. 7 and Table 2). pACEBac‐ADDomer is used as a backbone in this protocol for inserting DNA encoding for this E2EP3 epitope. The steps described in Basic Protocol 4 for purification of the vaccine candidate are used in this protocol.

### Materials

pACEBac‐ADDomer (Basic Protocol 4, step 4)
E2EP3 neutralizing epitope peptide (see Fig. 7 and Table 2)
DH10EMBacY cells (Geneva Biotech SARL, Switzerland)
Hi5 insect cells
Phosphate‐buffered saline (PBS)
EDTA‐free complete protease inhibitor (Roche, Switzerland)
High Q column (Bio‐Rad)

**Figure 8:**
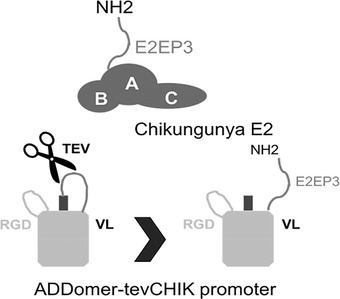
Native‐like peptide epitope presentation on ADDomer‐tevCHIK. Chikungunya envelope protein E2 consisting of three domains is shown (A, B, and C). E2EP3 (comprises the N‐terminus of E2 with the amino acid sequence NH2‐STDKDNFNVYKATRPYLAH). This E2E3P peptide, the major neutralizing epitope in the Chikungunya virus, is presented in an exposed native‐like conformation in ADDomer‐tevCHIK, with native‐like meaning that the N‐terminal serine residue of the epitope is accessible exactly as is the case in the Chikungunya viral envelope protein containing the major neutralizing epitope E2EP3. The N‐terminus was liberated by TEV protease cleavage at a specific site as indicated.

1 Insert E2EP3 into ADDomer^©^ to create ADDomer‐tevCHIK VLP vaccine candidate to combat Chikungunya infectious disease (Basic Protocol 4 steps 1‐4).
2 Apply steps 5‐12 of Basic Protocol 4 here to purify the Chikungunya vaccine candidate, by using the plasmid generated in step 1 of Basic Protocol 5 instead of pACEBac‐ADDomer.
3 At the N‐terminus of E2EP3 in ADDomer^©^, a site for a highly specific protease [tobacco etch virus (TEV) NIa] is included preceding the epitope. Thus, upon cleavage, generate a native‐like conformation comprising an exposed N‐terminal serine.
  Of note, negative‐stain EM analysis confirmed that the ADDomer dodecahedron stayed intact in spite of 60‐fold cleavage by TEV (Fig. 8; Vragniau et al., 2019).

4 Our approach is validated with immunization experiments. Sera of mice injected with uncut ADDomer‐tevCHIK did not yield E2EP3‐specific immunoglobulin titers when assayed against synthetic E2EP3 peptides in an enzyme‐linked immunosorbent assay (ELISA; Vragniau et al., 2019). In contrast, TEV NIa quantitatively cleaved ADDomer‐tevCHIK, exposing native‐like E2EP3 that elicited a very strong specific immunoglobulin G (IgG) response in ELISA when injected into mice, in agreement with the immune reaction that is observed in patients after Chikungunya viral infection.
5 An unrelated nanoparticle scaffold made of polylactic acid fused to a similar amount of E2EP3 peptide epitopes did not elicit a comparable immune response, indicating that the ADDomer^©^ scaffold itself may have an adjuvating effect.

## REAGENTS AND SOLUTIONS

### Hypotonic lysis buffer

Prepare 0.33× phosphate‐buffered saline (PBS, Thermo Fisher Scientific, cat. no. 10010023 supplemented with EDTA‐free cOmplete protease inhibitor (Roche, cat. no. 11873 580001; 1 tablet per 50 ml).

### Purification buffer gradient solutions

Prepare 10 mM HEPES (pH 7.4) containing 50 mM NaCl and 500 mM NaCl. Filter sterilize and store at 4°C up to 1‐2 months or longer. Just before purification, supplement with one EDTA‐free cOmplete protease inhibitor per 50 ml.

## COMMENTARY

### Background Information

Virus‐like particles (VLPs) are large protein assemblies that self‐assemble into structures mimicking naturally occurring viruses in shape and size. VLPs can induce strong immune responses but are not infectious, as they do not contain viral genetic material. VLPs are cornerstones of vaccine development. The first VLP‐based vaccine was the HBV vaccine HEPTAVAX‐B by Merck in 1981 (Zhao, Li, Yu, Xia, & Modis, 2013). Other examples include VLP‐based HPV vaccines such as Gardasil1 (Merck) and Cervarix1 (GlaxoSmithKline), which are produced in yeast and insect cells, respectively (Rodriguez‐Limas, Sekar, & Tyo, 2013).

There are important advantages when using VLPs in vaccine development and deploying them against emerging viruses. VLPs are considered safe due to their lack of genetic material, setting them apart from heat‐inactivated or attenuated live virions, which have intact genomes and could possibly revert again to replicating pathogens. Thus, VLPs provide a safe(r) research and application framework. VLPs may present major antigens and elicit strong immune responses, as they also contain repetitively arranged surface proteins with viral epitopes, similar to native viruses. VLPs were shown to induce strong B cell responses (Roldão, Mellado, Castilho, Carrondo, & Alves, 2010), but can also activate the cellular response by internalization in antigen‐presenting cells that activate CD4^+^ and CD8^+^ T cells (Chackerian, Lenz, Lowy, & Schiller, 2002; Cox et al., 2014; Wagner, Deml, Schirmbeck, Reimann, & Wolf, 1994). A major advantage is that VLPs can often be produced recombinantly, resulting in cost‐effective vaccines that are particularly beneficial for deployment in low‐income countries (Roldão et al., 2010).

A range of recombinant expression platforms are successfully used for VLP production, each with their own merits. These include bacteria (*E. coli*), yeast, insect cells, mammalian cells, plant expression systems, and more recently also in vitro cell‐free systems. *E. coli* has been used to produce several commercial VLP vaccines, for example against hepatitis (Hecolin1, Xiamen Innovax Biotech Co. Ltd.). Even though bacterial expression is cheaper and easier to handle, it lacks post‐translational modifications and sophisticated folding machinery that may be required for more complex VLPs. Yeast was used for the HBV vaccine Engerix‐B^Ⓡ^ (GlaxoSmithKline) and the HPV vaccine Gardasil^Ⓡ^. Alternatively, baculovirus/insect cell and mammalian systems that afford authentic post‐translational modifications including glycosylation and a sophisticated folding machinery can be more suitable for producing complex VLPs at high levels (Rodriguez‐Limas et al., 2013). Examples include the HPV vaccine Cervix^Ⓡ^ and the HBV vaccine GenHevac B^Ⓡ^ (Pasteur‐Mérieux Aventis), which were produced using these systems. The baculovirus insect cell system has the added advantage of operating in a regular lab safety level environment (BSL‐1) due to its narrow host range (insect cells) largely eliminating any biological hazard for the user.

VLPs such as the influenza VLPs described here may comprise several antigenic components (e.g., HA, NA, M2) in addition to the component forming the particle (M1). The MultiBac baculovirus insect cell expression system is particularly suited for co‐expressing multiple genes from a single baculovirus (Berger et al., 2004; Fitzgerald et al., 2006; Fitzgerald et al., 2007). This enables not only the co‐expression of multiple antigenic components to produce potentially superior VLPs that mimic live viruses, but also enables provision of additional factors, for example specific chaperones to facilitate production of the product of choice.

The MultiBac baculoviral genome is propagated as a single‐copy bacterial artificial chromosome in specialized *E. coli* strains (Luckow & Summers, 1988, Berger et al., 2004; Fitzgerald et al., 2006), which greatly simplifies integration of genes encoding the components of VLPs. The very strong polyhedrin (polh) and p10 late viral promoters (Belyaev & Roy, 1993) used for driving recombinant expression often result in excellent expression levels on the order of magnitude observed here for ADDomer^©^. Nonetheless, if needed, any other viral or cellular promoter can be utilized by adjusting the modular transfer plasmids (Table 1; Fitzgerald et al., 2006).

Virus performance and sample production can be easily monitored by co‐expressing fluorescent proteins as reporters (e.g., mCherry as in this protocol). These reporter genes are easily co‐integrated into the MultiBac baculoviral genome, in addition to the genes encoding the components of a VLP of choice. Monitoring the reporter boosts reproducibility and enables the development of customized, controlled, and standardized operating protocols, which are essential prerequisites for large‐scale manufacturing (Monteiro et al., 2016).

### Critical Parameters and Troubleshooting

#### Restriction sites

The production of MultiBac‐based VLP array includes the step for generation of modular expression cassettes, and specific restriction enzymes are part of the protocol (e.g., *Pme*I, *Spe*I, *Avr*II, *Bst*Z17I and/or *Nru*I). Ideally, these are eliminated from the ensemble of genes relevant for the production of the VLP of choice. This is best accomplished by ordering the synthetic genes with this requirement in mind.

#### In vitro Cre recombination of Acceptor and Donor derivatives

It may be planned to utilize in vitro Cre recombination (Fitzgerald et al., 2006) to fuse gene expression cassettes for multicomponent VLP production. Each Acceptor and Donor of the MultiBac system (Table 1) has a unique resistance marker. The resistance marker combinations resulting from Cre fusion enable selection of desired fusions following transformation of the Cre‐fusion reactions into *pir* ^−^ strains (e.g., TOP10 or DH5α). Plasmids resulting from incomplete fusion will then be eliminated based on the conditional R6Kγ origin of replication, which is not recognized in *pir^−^* cells. For example, multigene plasmid assembly can be performed in a single reaction with, e.g., two Donor derivatives (with different resistance markers) and one Acceptor, and the fusions can be monitored on agar plates including the three respective antibiotics. We have found this method often preferable and more versatile compared to classical or ligation‐independent cloning methods to assemble multiexpression constructs.

#### Integration of multiple copies of same Donor

It can, however, occur (rarely) that several copies of a Donor are combined with one Acceptor during the in vitro Cre‐fusion step. This can be verified by using restriction mapping based on predictions from the software Cre‐ACEMBLER, which we developed and which is available online (Nie et al., 2016; Sari et al., 2016). Often, the resulting multiexpression construct can nonetheless be used for MultiBac‐based expression, sometimes even in a beneficial manner, as multiple insertion of the same gene may result in increased expression.

#### Using both loxP and Tn7 sites for integration into MultiBac bacmid

When one wants to use both the *loxP* and the Tn7 sites of the MultiBac baculoviral genome for integration, it is recommended to first occupy the *loxP* site by transforming the corresponding Donor into DH10MultiBac^Cre^ cells. This was the procedure applied here in VLP‐factory^©^ for providing the genes encoding H1N1 M1 and mCherry. Then, a blue positive clone comprising the composite bacmid is picked in the blue/white screening for further processing, and competent cells prepared. In the next step, Acceptor derivatives comprising gene(s) of choice (here HA wild‐type and ISD mutants) are transformed into these competent cells for insertion of Acceptor derivatives, or Acceptor‐Donor fusions, into the Tn7 site (Fig. 1). Tetracycline should always be present when competent cells are prepared, to maintain the Tn7 transposition factors encoded by a tet‐resistant helper plasmid.

#### Transfection and cell proliferation

Occasionally, when following the standard MultiBac protocol (Bieniossek et al., 2008; Fitzgerald et al., 2006), the initial V_0_ and V_1_ viruses may have low titers, which may happen to new or inexperienced users of the system. In this case, cell division initially can require a longer time before proliferation arrest, indicating wholesale infection of all cells in the culture. This issue can be overcome by repeating the experiment, providing a larger virus volume used for infection. If no proliferation arrest is observed even with more virus added, it is recommended to repeat the initial transfections. Conversely, immediate cell proliferation arrest can be observed if the virus titers are too high. Then, the cell culture should not be used for virus production, as defective virus can accumulate and give diminishing target protein expression. In this case, we suggest repeating the experiment with less virus added.

#### Protein production levels

Insect cells may age over time, and the protein expression level can be affected by cell aging. To forestall this, it is recommended to change to a fresh cell culture thawed from frozen stocks every 8‐12 weeks. Importantly, the recommended shaker flask volumes should be followed closely to ascertain proper aeration (Fitzgerald et al., 2006). For cell adaptation from frozen stock or monolayer to flasks, it is advised to let them divide at least once before infecting them with the virus. If no target protein expression is observed, infection can be repeated, varying virus volumes at the same time. A new initial transfection with the same expression construct should also be started, to avoid losing time given that the protocols for insect cell expression are measured in days (rather than hours as for expression in *E. coli*).

### Understanding Results

The MultiBac expression system we use here has proven its aptitude for successful production of complex protein biologics including VLPs. Our MultiBac‐based VLP‐factory™ can be used to produce many different enveloped VLPs in addition to the influenza VLPs described here. The versatility of the approach combined with the high yields obtained enable rapid testing of many variants of a given VLP, as exemplified here for immune‐suppressive domain mutations in HA glycoproteins of the influenza virus, helping to identify suitable, ideally hyper‐immunogenic candidates for vaccine development. In a similar vein, VLP‐factory™ or a corresponding MultiBac‐based system may be useful to develop next‐generation vaccines against SARS 2 coronavirus, the causative agent of the COVID‐19 pandemic.

**Figure 9:**
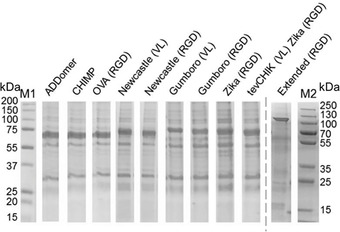
Genetically encoded multiepitope display by ADDomer^©^. Coomassie‐stained SDS‐PAGE of ADDomer^©^ VLPs is shown displaying a range of epitope arrangements (see Table 2). CHIMP, chimpanzee; OVA, melanoma model epitope; CHICK, Chikungunya major neutralizing epitope; tev, Tobacco Etch Virus NIa proteolytic site; M1, M2 are molecular weight markers (sizes in kDa). The variability of the migration pattern of the ADDomer band reflects the different sizes of the constructs.

We also present here a non‐enveloped type of synthetic VLP, the ADDomer^©^. The plug‐and‐play setup enables simple insertion of entire libraries of immunogenic epitopes, in multiple copies, from a wide range of human and livestock viral pathogens. ADDomer^©^ has a remarkable capacity to accommodate very large multiepitope insertions in the loci that we engineered (Fig. 9; Table 2). For example, we successfully inserted multiple copies of immunogenic epitopes arranged in a string, derived from Gumboro virus. Gumboro causes infectious bursal disease which affects young chickens, turkeys, and ducks, causing considerable economic damage especially in developing countries. Both VL and RGD loops accommodated the epitope strings without detrimental effect on dodecahedron integrity. In a different experiment, we inserted an artificial epitope comprising more 200 amino acid residues in the RGD loop, resulting in fully functional particles, demonstrating that long sequences, maybe even representing structured domains, can be efficiently displayed at high density on the ADDomer^©^.

### Time Considerations

The time needed for the cloning strategy and then cloning the genes of interest into multigene transfer vectors can vary based on the number of subunits present in the VLPs of choice, the strategy chosen, and the experience of the researcher. It can take several weeks. This time can be considerably shortened by ordering synthetic DNAs now offered at very competitive prices.

Preparation of DH10VLP‐factory™ aliquots requires 2 to 3 days, and then transformation of the transfer plasmid into one of the DH10VLP‐factory™ aliquots and composite bacmid isolation will take another 2 to 3 days.

Insect cell cultures should be monitored every 24 hr for their density and proliferation, which is probably the most tvime‐consuming part of this protocol. Most of the manipulations of the cell cultures must be implemented in a sterile hood. After initial transfection, about 60 hr later, V_0_ virus can be harvested. Afterwards, virus is amplified and VLPs produced. The time needed for all cells to be infected and reach the point of proliferation arrest so that V_1_ virus can be collected is 48‐60 hr. After 48‐60 hr, more than 95% of cells are infected with the VLP‐producing baculovirus. Cell harvesting time also need to be taken into consideration, as cell viability usually starts dropping 72 hr after proliferation arrest. Also, for the majority of proteins, there is an expression plateau between 72 and 84 hr post infection. As we described here for VLP‐factory™ production of flu VLP arrays, the logistics for each round encompassed production in batches of 25 different VLPs, each in 50 ml of cell culture in a 250‐ml Erlenmeyer flasks. For each individual VLP produced, V_0_ and V_1_ collection should be done separately to forestall cross contamination. This can take several hours/days to complete, and needs to be planned properly in advance.

Analyzing the expression of VLPs typically requires SDS‐PAGE, given the usual high expression of the target proteins. Preparing and running SDS‐PAGE and staining of the gel with a protein dye will require on the order of 3‐4 hr. Typical yields per liter of culture vary between different VLP constructs, but we have obtained up to 500 mg/L.

Analysis of the VLPs for functionality, in particular as candidate vaccines, will strongly depend on the animal models required and the disease indications, and will vary from case to case.

### Author Contributions

**Duygu Sari‐Ak**: Conceptualization; Methodology; Visualization; writing‐original draft; writing‐review & editing. **Joshua Bufton**: Investigation; Resources; Visualization; writing‐review & editing. **Kapil Gupta**: Data curation; Methodology; writing‐review & editing. **Frederic Garzoni**: Conceptualization; Investigation; Methodology; Project administration; writing‐review & editing. **Daniel Fitzgerald**: Conceptualization; Investigation; Methodology; Project administration; Supervision; Validation; Visualization; writing‐review & editing. **Christiane Schaffitzel**: Conceptualization; Investigation; Methodology; Project administration; Supervision; Visualization; writing‐review & editing. **Imre Berger**: Conceptualization; Funding acquisition; Investigation; Methodology; Project administration; Resources; Supervision; Validation; Visualization; writing‐original draft; writing‐review & editing.

### Conflict of Interest

I.B., F.G. and D.F. declare conflict or interest related to this correspondence. I.B. and F.G. are shareholders of Imophoron Ltd, Imophoron owns patents and trademarks related to ADDomer and its applications. I.B. and D.F. are shareholders of Geneva Biotech SARL. Geneva Biotech owns patents related to the MultiBac system and its applications including VLP‐Factory.

## Corrections

In this publication, author's conflict of interest and data availability statement have been added.

The current version online now includes this information and may be considered the authoritative version of record.

## Data Availability

Data sharing not applicable–no new data generated.
